# Preparation and Evaluation of Oxaliplatin Thermosensitive Liposomes with Rapid Release and High Stability

**DOI:** 10.1371/journal.pone.0158517

**Published:** 2016-07-14

**Authors:** Chunying Zeng, Fanglin Yu, Yang Yang, Xiaohui Cheng, Yan Liu, Hui Zhang, Shiqing Zhao, Zhenbo Yang, Mingyuan Li, Zhiping Li, Xingguo Mei

**Affiliations:** 1 Pharmacy Department, Fu Xing Hospital Capital Medical University, Beijing, China; 2 Pharmaceutical Department, Beijing Institute of Pharmacology and Toxicology, Beijing, China; 3 Pharmacy Department, No. 261 Hospital of PLA, Beijing, China; Wuhan University of Science and Technology, CHINA

## Abstract

Oxaliplatin (OXP) was reported to show low anti-tumor activity when used alone and to display side effects; this low activity was attributed to high partitioning to erythrocytes and low accumulation in tumors. Thermosensitive liposomes (TSL) were considered able to specifically deliver drugs to heated tumors and to resolve the OXP distribution problem. Regretfully, TSL encapsulating doxorubicin did not demonstrate significant improvement in progression-free survival. Drug release below 41°C and significant leakage were considered major reasons for the failure. The purpose of this study was to acquire OXP TSL with rapid release at the triggered temperature and high stability at body temperature and at storage temperatures. A small quantity of poloxamer 188 was introduced into the TSL formulation to stabilize the encapsulated drug. It was shown that the addition of poloxamer 188 had no influence on the TSL characteristics. More than 90% of OXP was released within 10 min at 42°C, and less than 15% was released within 60 min at temperatures below 39°C. TSL were stable at 37°C for 96 h and at 4°C for 6 months. The anti-tumor activity of TSL at the dose of 2.5 mg/kg was certified to be equal to those of OXP injection and non-thermosensitive liposomes (NTSL) at the dose of 5 mg/kg, and significant improvement of tumor inhibition was observed in TSL compared with injection and NTSL at the same dose. It was also shown from the histological transmutation of tumors that TSL had stronger anti-tumor activity. Therefore, it could be concluded that TSL composed of a proper amount of poloxamer had rapid release and high stability, and OXP TSL would be anticipated to exert prominent anti-tumor activity in the clinic.

## Introduction

Oxaliplatin (OXP) is an anti-cancer agent that belongs to the third generation of platinum compounds [1] and is now approved as first-line chemotherapy in combination with 5-fluorouracil for the treatment of advanced colorectal cancer in several major European countries [2]. However, low anti-tumor activity *in vivo* is shown for OXP used alone [3, 4]. Moreover, a few side effects, including acute dysaesthesias and cumulative peripheral distal neurotoxicity, are further displayed for OXP, despite its better tolerability in comparison with other Pt compounds. These unsatisfying actions may be attributed to its high partitioning to erythrocytes, low accumulation in tumor tissues after intravenous administration [5] and thereby the improved drug dose [6]. Therefore, there is an imminent need to develop an effective way to overcome these problems [5].

The most satisfying approach resolving these puzzles is to deliver a high concentration of the drugs to tumor tissues, and numerous targeted drug delivery systems [2, 3, 5] have been developed. Of these, the liposomal drug delivery system can alter the pharmacokinetics and biodistribution of the encapsulated drug and is believed to be an advanced and promising system for selective delivery of anti-cancer drugs to solid tumors [5]. Liposomes are small and spherical vesicles with a phospholipid bilayer structure, similar to cells, and are reported to fuse easily with cells, thereafter deliver drugs into the cytoplasm, so they are deemed to be the most effective drug carriers [7]. Until now, a few liposomal products such as DEPOCYT, DEPRDUR, EXPAREL, DAUNOXOME, DOXIL and DOXORUBICIN have been approved for market [6]. However, their clinical use is limited by rapid opsonization and uptake mediated by the cells of the mononuclear phagocyte system (MPS) [8]. Modification of the liposomal surface with high molecular hydrophilic polymers, such as polyethylene glycol (PEG), is considered effective for protecting liposomes from interactions with blood proteins, blood cells or MPS cells, and thus, extending their blood circulation time [8]. Side effects of cytotoxic drugs, such as doxorubicin, have been proven to be reduced markedly by encapsulating drugs in PEGylated liposomes [9]. Despite the preferential accumulation at tumor site, and thereby lower toxicity compared with free drug, there was no improved effectiveness exhibited in these clinically approved liposomal formulations [10] because efficacious release of drugs from these vesicles is absent. So, it is a prerequisite to release drugs effectively once liposomes accumulate in the target site [9].

To enhance drug release, liposomes sensitive to stimuli, such as pH [11], temperature [12], high intensity focused ultrasound (HIFU) [13] and microwaves (MWs) [14], are focused. In particular, thermosensitive liposomes (TSL) have received considerable attention because of the controlled and increased drug release by external hyperthermia [15]. TSL are able to release encapsulated actives when the triggered temperature is above or near their phase transition temperature (Tm), where a transition of the lipid membrane from gel to liquid crystalline phase happens [16]. This temperature-triggered release can be controlled spatially and temporally by steering the heating focus and heating power externally. Furthermore, application for regional or localized heating of even deep-seated tumor tissue is also well established in clinical practice [17]. TSL are considered prospective because hyperthermia treatment (HT) is always no more than 42°C, which is well accepted as mild hyperthermia and has been applied to treat solid tumors in the clinic [18]. Meanwhile, the drug release triggered by mild temperature (approximately 42°C) has been confirmed both *in vitro* and in mice [18, 19]. Unfortunately, the effectiveness of TSL in clinical trials is not as good as expected; Celsion Corp. announced that its late-stage drug ThermoDox in combination with radiofrequency ablation (RFA) failed to be effective because significant improvement in progression-free survival could not be demonstrated [20]. Drugs released from the vesicles below 41–42°C and leaking significantly into the blood circulation before the accumulation of vesicles in the tumor tissues were considered the major reasons leading to the failure of ThermoDox [19]. To prevent serious leakage and early release of encapsulated drug before arriving at the target site, lots of modifications to TSL, such as preparing high temperature-triggered TSL with a Tm of approximately 44°C [9] and nanogel thickened TSL [21], have been carried out to stabilize the liposomes. However, these modifications always reduced the release rate, and a long time was required for complete release; this extended release time does not match with the therapy time of HT in clinic.

In this paper, to obtain rapid release once discharge from TSL is triggered, poloxamer, a type of thermosensitive material, was added into the TSL formulation, with OXP as model drug. The results showed that TSL with poloxamer exhibited the proper release time at the triggered temperature and high stability at both body temperature and storage temperature. The significantly improved tumor inhibition of TSL with poloxamer was also shown in nude mice.

## Materials and Methods

### Materials

The lipid bilayer components, 1,2-dipalmitoyl-sn-glycero-3-phosphatidylcholine (DPPC), monostearoylphosphatidylcholine (MSPC), 1,2-distearyl-sn–glycerol-3- phosphoethanolamine-N-[methoxy-(polyethyleneglycol)-2000] (DSPE-PEG2000) and cholesterol were purchased from Shanghai Advanced Vehicle Technology Pharmaceutical LTD (Shanghai, China). Poloxamer 188 was purchased from Beijing Fengli Jingqiu Commerce and Trade Co., Ltd. OXP was obtained from Jie Rui pharmaceutical company (Jiangsu, China). OXP for injection was purchased from Heng Rui pharmaceutical company (Jiangsu, China). All these reagents were of analytical grade.

Lewis Lung Cancer Cell (LLCC) was purchased from the Cell Resource Centre (IBMS, CAMS/PUMC). Male Nu/Nu nude mice (weighing 18–20 g) were purchased from Vital River Laboratories (Beijing, China). Mice were all housed in ventilated cages with free access to food and water under standardized conditions (artificial lighting from 7:00 am to 7:00 pm, temperature at 24±1°C and relative humidity at 50±10%). Mice were acclimatized to laboratory condition for one week before the experiment. Meanwhile, a dim red light was used for mice treated during the dark phase. All surgery was carried out under sodium pentobarbital anesthesia and all efforts were made to relieve suffering. All animals were handled in research according to the code of ethics laid down by the Animal Care and Use Ethics Committee of Academy of Military Medical Sciences.

### Preparation of liposomes

OXP TSL were composed of DPPC, MSPC, poloxamer 188 and DSPE-PEG2000 (85:9.5:0.5:5, molar %). OXP non-thermosensitive liposomes (NTSL) were composed of HSPC, cholesterol and DSPE-PEG2000 (85:10:5, molar %). All liposomes were prepared according to the reported method with some modifications [22, 23]. Briefly, the lipids were dissolved in chloroform, the solvent was evaporated under vacuum by a rotator RE-2000 (Ya Rong Biochemical Instrument Factory, China) and a thin film was formed. The formative film was then hydrated using OXP solutions with or without poloxamer 188 by rotating the flask, and multilamellar vesicles were obtained after approximately 60 min at room temperature. The suspensions were then extruded through a polycarbonate membrane (200 nm pore size) using an extruder machine (EmulsiFlex-C3, Avestin, Canada). Unencapsulated OXP and poloxamer was removed by dialysis. Thus, two types of liposomes with homogeneous shape and uniform size were acquired.

### *In vitro* characterization of OXP liposomes

#### Morphology

The morphology of liposomes was observed by transmission electron microscopy (TEM). Liposomes were diluted with distilled water to a certain concentration and dropped on a copper grid to form a dry film at room temperature. Then, liposomes were negatively stained with 2% phosphotungstic acid. The sample was air-dried at room temperature once again and then observed using TEM (JEM-1010, JEOL Ltd., Tokyo, Japan).

#### Particle size

Particle size and population distribution of TSL and NTSL were determined by photo cross correlation spectroscopy (Nanophox, Sympatec GmbH, Germany). The laser intensity was adjusted to 50%-60%, the cross correlation mode was used, and the analysis was operated at 25°C. The measurements were conducted in triplicate. The results were reported as the mean value of 50% particle distribution.

#### Differential scanning calorimetry

The phase transition temperatures of TSL and NTSL were determined with differential scanning calorimeter (DSC, TA Instruments, USA). The temperature was increased from 30°C to 50°C with a speed of 2°C/min.

#### Encapsulation efficiency

Encapsulation efficiency (EE) of OXP in liposomes was determined by a modified ultra-filtration centrifugation method [24]. Briefly, 100 μL of liposomes was diluted to 5 mL with distilled water, added into the ultra-filter (Vivaspin2; Sartorius Biotech, USA) and then centrifuged at a speed of 1,2000 rpm for 10 min. The filtrate containing free OXP was collected and stored at 4°C until analysis. Another 100 μL of liposomes was added into 200 μL of 10% (W/V) triton to destroy the structure of liposomes completely and form a uniform solution containing free OXP.

The amount of free OXP in filtrate and the total amount of OXP encapsulated and unencapsulated in liposomes were all determined by HPLC. The separation was performed on a normal phase silica column (Megres C18, 4.6×250 mm, 5 μm, Hanbon Sci. &Tech, Jiangsu, China). The mobile phase consisted of methanol and water (4:96, V/V) and was pumped at a flow rate of 1.0 mL/min. The detection wavelength was 210 nm, and the injection volume was 20 μL.

EE was obtained according to Eq 1, where OXP_total_ indicates the amount of OXP in the whole liposome suspension, and OXP_free_ indicates the amount of OXP unencapsulated in the liposomes.

$$Encapsulation\;efficiency\;(\%)=\frac{OXP_{total}-OXP_{free}}{OXP_{total}}\times 100\%$$ (1)

### Temperature triggered release *in vitro*

The drug release studies were carried out at 37°C, 39°C, 41°C and 42°C, respectively. Briefly, 2 mL of liposomes was diluted to 20 mL with 5% glucose solution, stirred and heated at different temperatures. At various time intervals, certain volumes of samples were withdrawn and separated immediately with ultra-filter under centrifugation [16]. The OXP concentrations in the filtrates were determined by HPLC. The cumulative releases were calculated according to Eq 2. The *in vitro* release curves were plotted with the cumulative percentage of drug released as a function of time (minutes).

$$Cumulativerelease(\%)=\frac{\left(V\times C_{t}+V_{r}\times \sum C_{m}\right)}{Dose}\times 100\%$$ (2)

Where V is the volume of dissolution medium, C_t_ represents the measured concentration of OXP in the filtrate at time t, ∑C_m_ denotes the sum total of the previous concentrations, V_r_ corresponds to the volume of samples removed for analysis, and dose is the amount of OXP added into the release medium.

### Short-term and long-term stability

TSL and its 50-fold dilutions with 5% glucose solution and cell culture media (MEM) containing 10% fetal bovine serum (FBS) were kept at 37°C, and parameters of these samples, including particle size, pH and EE, were monitored daily for short-term stability. To evaluate the stability of TSL in the long term, TSL was kept at 4°C, and indexes comprising particle size, pH and EE were examined regularly during a period of 6 months. The stability of TSL and its dilutions was also monitored with a Turbiscan Lab^®^ Expert (Formulaction, L’Union, France), an innovative analytical instrument able to determine the small changes of colloidal systems. To obtain these data, the samples were placed into cylindrical glass tubes and then submitted to Turbiscan Lab^®^ Expert at predesigned time points. The stability was evaluated with delta transmission and delta backscattering as indexes [25].

### *In vivo* anti-tumor efficacy

The anti-tumor efficacy was evaluated in LLCC tumor-bearing male nude mice. Briefly, the mice were inoculated subcutaneously at the flank region with 200 μL of cell suspension containing 2×10^7^ LLCC, and the tumors were allowed to grow until the volume was approximately 200 mm^3^. The mice were randomly divided into six groups (12 mice per group), and mice in each group were treated every four days by tail vein injection with 5% glucose solution (Control group), OXP solution (5 mg/kg), TSL (2.5 mg/kg, 5 mg/kg and 10 mg/kg) and NTSL (5 mg/kg). The injection volume was approximately 200 μL, which was calculated from the weight of mice and the administration dosage. The tumor regions were heated using a homemade hyperthermia device connected with a thermostatic circulator (HX-1050, Beijing Boyikang Laboratory Apparatus Co., Ltd., China) at 42°C for 30 min after injection. The tumor volume of mice was measured daily and calculated based on Eq 3, where “a” and “b” indicate the length and width of the tumor, respectively. The animals were also weighed and observed daily during the experimental period to monitor the health condition of animals and evaluate the preliminary toxicity of the formulations. After 12 days, the mice were sacrificed, and the tumors were excised and weighed. The tumor inhibition rate was calculated according to Eq 4, where M represents the average weight of tumors in the control group and M_t_ represents the average weight of tumors in each treated group. The excised tumors were fixed in formalin and sliced after paraffin embedding. The slice was stained with hematoxylin and eosin (H&E) for histopathological analysis.

$$Tumor\;volume=\frac{a\times b^{2}}{2}$$ (3)

$$Tumor\;inhibition\;rate\;(\%)=\left(1-\frac{M_{t}}{M}\right)\times 100\%$$ (4)

### OXP distribution in plasma and tumors

The OXP distribution in plasma and tumors was also evaluated in LLCC tumor-bearing male nude mice. The mice were randomly divided into six groups (3 mice per group), and mice in each two groups were treated every four days by tail vein injection with OXP solution, TSL and NTSL respectively at a dose of 5 mg/kg for 12 days. In one of the two groups given the same formulation, the tumor regions were heated at 42°C for 30 min after injection, and the tumors in the other group were treated without heat. Then, about 1 mL venous blood sample was collected from anesthetized animals in each group, thereafter the mice were sacrificed and the tumors were removed. All blood samples were centrifuged immediately at 4000 rpm for 10 min, and the plasma was obtained by collecting the supernatant. All of the excised tumors were weighed and homogenized. One hundred microliters of 6% (V/V) perchloric acid in water was added into 200 μL of the plasma or the tumor homogenate to precipitate the proteins, and the supernatants for analysis were collected after centrifugation.

The content of OXP in supernatants was determined with HPLC. Analysis was performed on a Venusil MP-C18 (5 μm, 250 × 4.6 mm, Agela Technologies, Tianjin, China) at room temperature, and the mobile phase contained acetonitrile– 0.1% (V/V) acetic acid in water (2:98, V/V) and was pumped at a flow rate of 1.0 mL/min. The detection wavelength was 250 nm and the injection volume was 50 μL.

### Statistical analysis

All data were expressed as the mean ± standard deviation (SD) unless specifically outlined. Student’s t-test or one-way analyses of variance (ANOVA) were performed for the statistical evaluation of data. Differences between groups were considered statistically significant when the probability (*p*) was less than 0.05.

## Results and Discussion

### Characteristics *in vitro*

**Fig 1:**
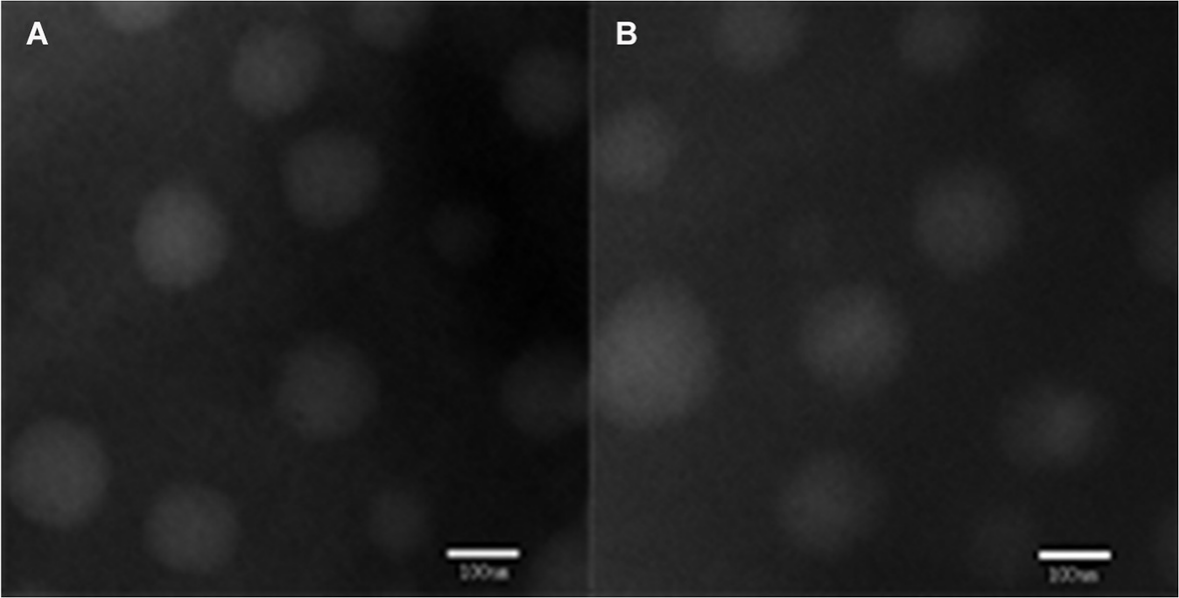
Transmission electron micrographs of TSL (A) and NTSL (B).

**Fig 2:**
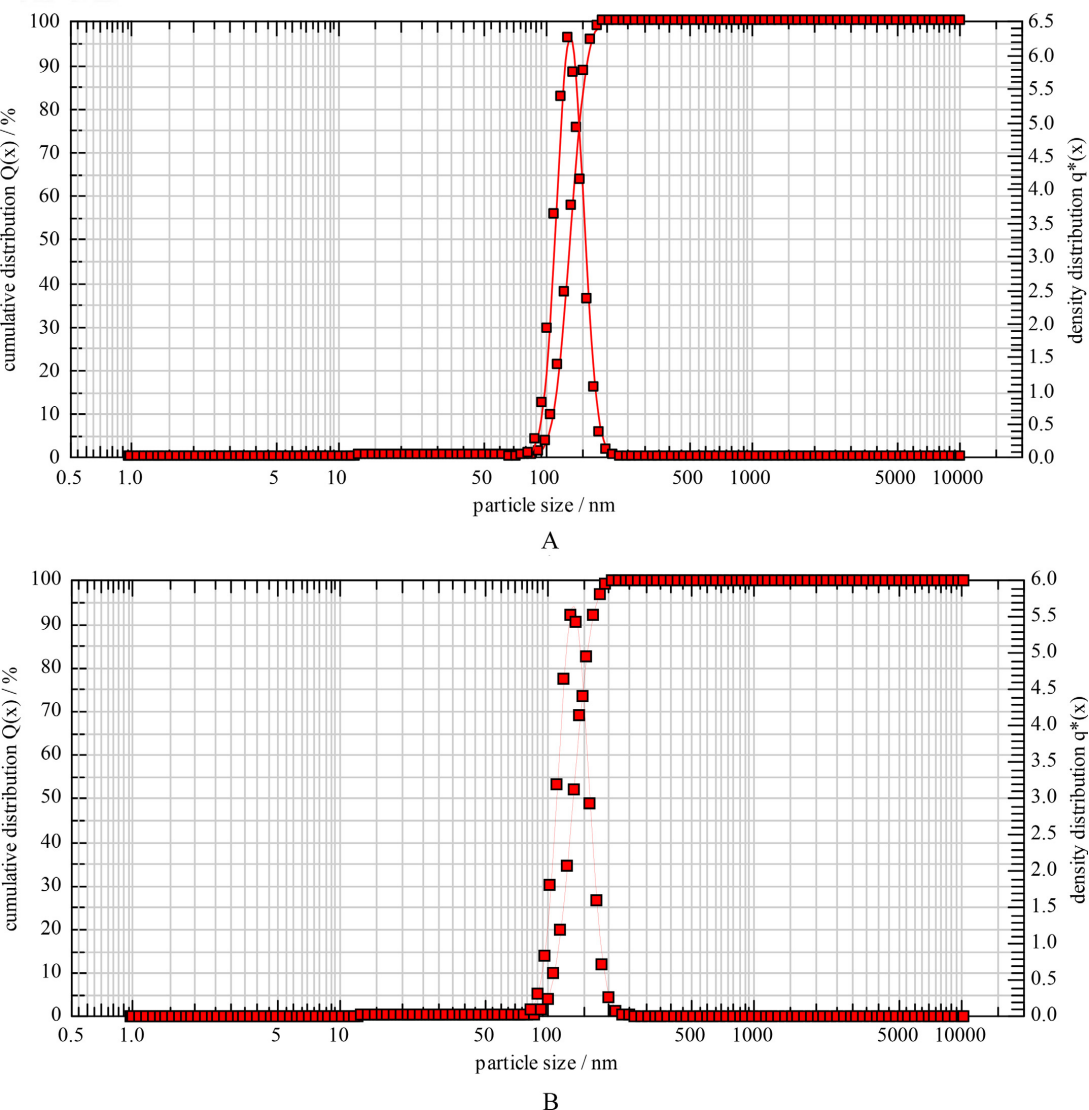
Particle size distribution of TSL (A) and NTSL (B).

**Fig 3:**
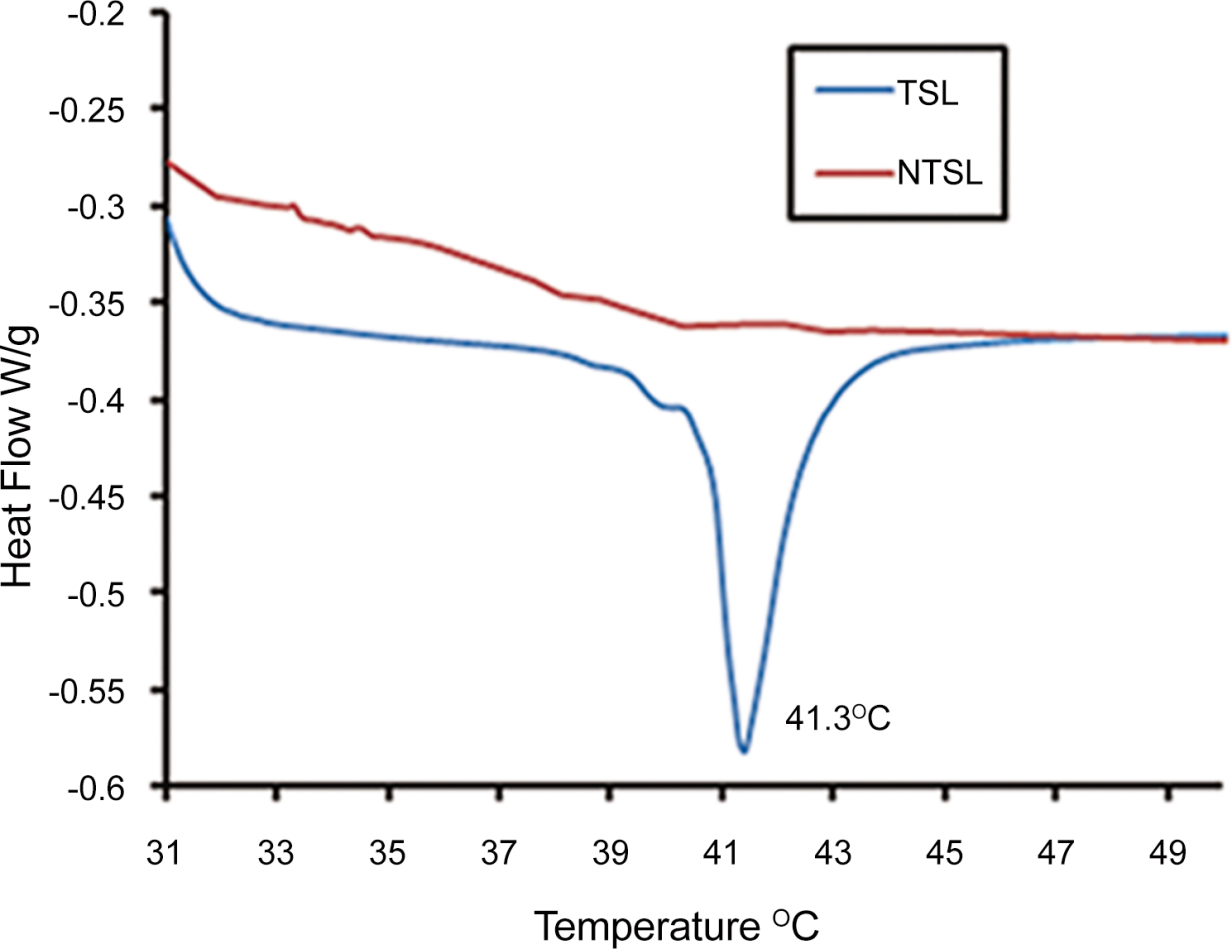
DSC thermographs of TSL and NTSL.

Two types of liposomes, namely TSL and NTSL, were fabricated both via the film evaporation/extrusion method. The liposomes were all regularly round and there was no morphologic difference between TSL and NTSL (Fig 1). The diameters of TSL and NTSL measured with photo correlation spectroscopy were 130.5±1.2 nm and 132.6±0.9 nm (*p*>0.05), respectively (Fig 2), which was consistent with the results obtained from TEM. The EE of TSL and NTSL were 91.2% and 90.5%, respectively. The zeta potentials of TSL and NTSL were -25.6±0.9 mV and -5.8±0.3 mV, respectively. The DSC data in Fig 3 displayed that there was a sharp transformation in curves of TSL between 40°C and 43°C and that the Tm was 41.3°C. These data suggested that TSL made in this paper had the optimal transition temperature accordant with the temperature in HT [16] and that the addition of poloxamer 188 had no effect on the Tm, as reported in the literature [26]. There was no phase transition observed within 30–50°C for NTSL.

### Temperature triggered release *in vitro*

**Fig 4:**
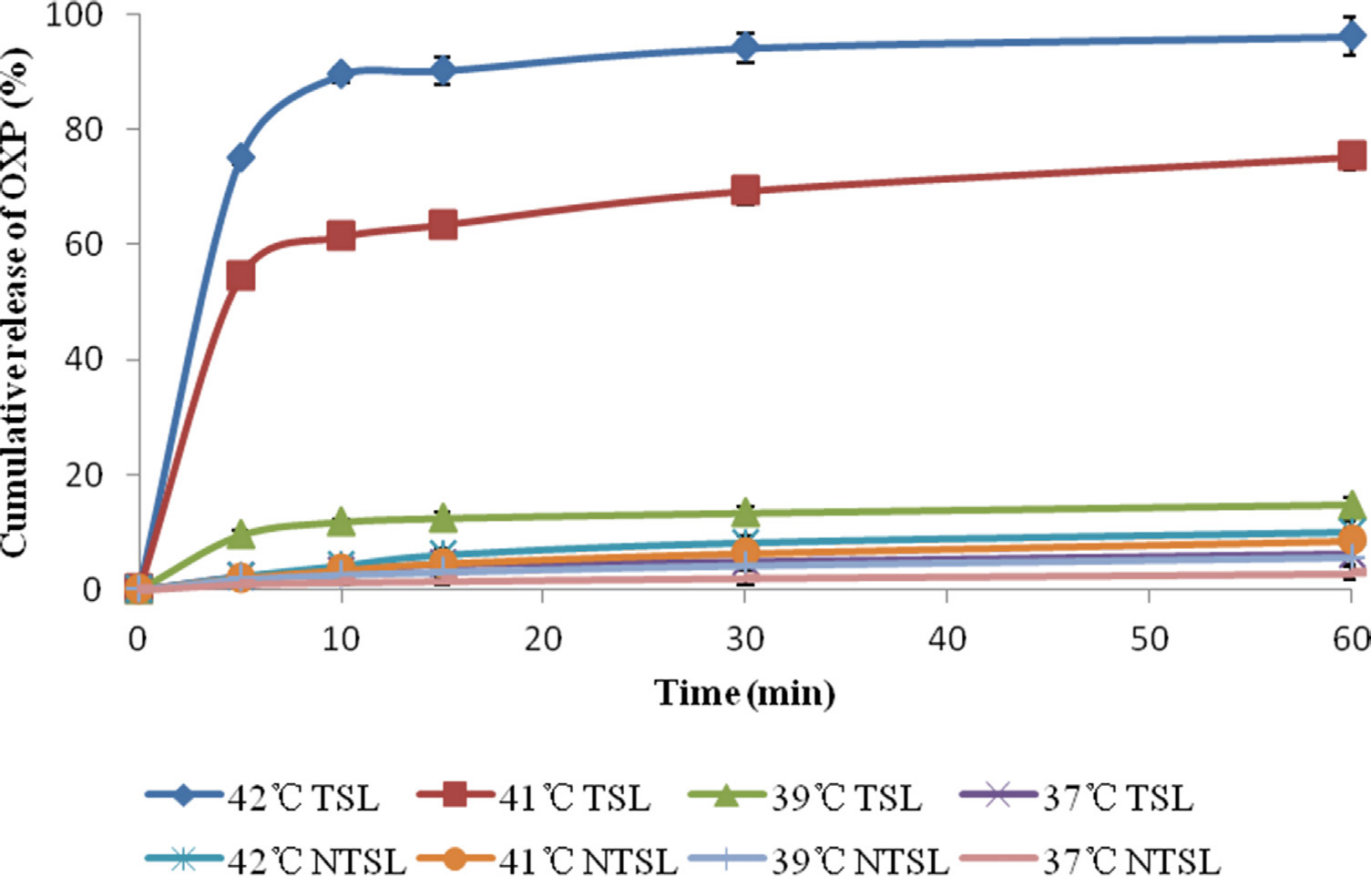
Temperature-triggered release profiles of OXP from TSL and NTSL at 37°C, 39°C, 41°C and 42°C, respectively.

The release of OXP from TSL and NTSL at 37°C, 39°C, 41°C and 42°C was depicted in Fig 4. It was shown that the release of OXP was very slow and that no more than 15% OXP was released at 60 min from TSL when the triggered temperature was below 39°C. The release of OXP accelerated markedly when the triggered temperature was near or above the Tm, and the cumulative release of OXP had reached 90% at 10 min when the triggered temperature exceeded Tm. However, the triggered temperature had almost no effect on the release of OXP from NTSL because the cumulative release of OXP was less than 15% at 60 min no matter what the temperature was. In summary, the release of OXP from TSL was temperature dependent while the release of OXP from NTSL was hardly affected by the triggered temperature, that is, TSL was thermosensitive while NTSL was insensitive to temperature.

Poloxamer is an amphipathic nonionic triblock copolymer composed of a central hydrophobic polypropylene oxide chain with two hydrophilic polyethylene oxide (i.e., PEG) chains [26, 27]. This specific chemical structure allows poloxamer to form micelles, self-organize and transform into viscous gel [28]; that is, poloxamer 188 is thermosensitive, and its low-viscous solution at low temperature can transform into viscous gel when the temperature rises above the sol-gel transition temperature (T_gel_) [29]. T_gel_ of poloxamer in situ gel depends on the concentration and the molecular weight of poloxamers and is commonly no more than 37°C [21]. Poloxamer 188 is considered safe and has been approved for application in injections by the Food and Drug Administration (FDA) [30]. As above, poloxamer 188 was added into the formulation to stabilize the liposomes utilizing its gel transition property. It was shown in Fig 4 that the addition of poloxamer had almost no influence on drug release because OXP was completely released within 10 min, and there was almost no difference in the release rate compared with TSL without poloxamer as previously reported [2, 16, 31]. This limited release-retarding ability of poloxamer might be due to its small quantity added, small size and strong hydrophilicity. However, the stabilization role of poloxamer 188 on TSL was apparent because no more than 15% of OXP was released from liposomes at body temperature during 60 min.

Ultrafast triggered release of drugs within 20 s [32] and slower release within one week from TSL [21, 33] have been reported. TSL with ultrafast triggered release were considered unstable because serious drug leakage was always observed, indicating that most of the drug couldn’t be delivered to the specific target site and that TSL with ultrafast triggered release would have poor effectiveness in the clinic [32]. On the other hand, TSL with a long release period were also thought undesirable because HT was always conducted within 45 min [20]. So, a release period of about 10 min, as shown in this paper, was considered optimal.

### Stability

The most promising liposomal system with desirable efficacy was always characterized by slow leaking and high stability as previous literature reported [34]. Regretfully, until now, liposomal formulations have been challenged by their instabilities in aqueous dispersions, such as liposome aggregation in long-term storage and encapsulated drug leakage [35, 36]. Thus, the evaluation of drug leakage and liposome aggregation or sedimentation during storage is very important to obtain an optimal liposomal drug delivery system. These indexes were also detected for TSL and diluted TSL described in this paper.

**Fig 5:**
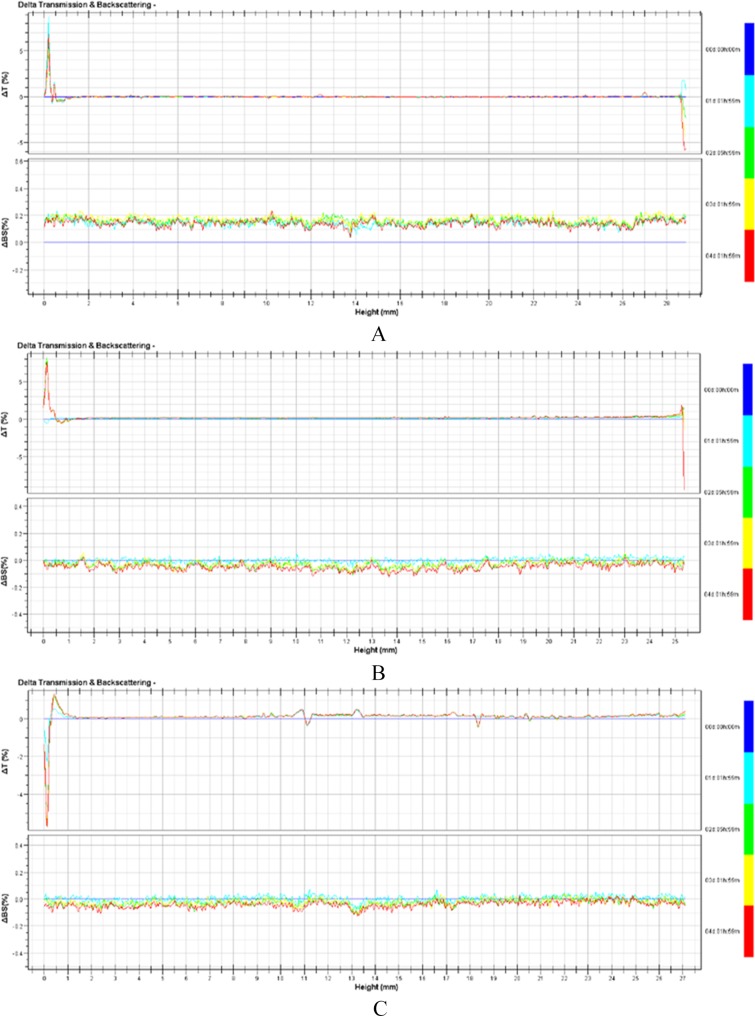
Variation profiles of transmission and backscattering for TSL and diluted TSL in the short-term stability study. A: TSL; B: TSL diluted 50-fold with 5% glucose solution; C: TSL diluted 50-fold with MEM culture media containing 10% FBS.

The results of TSL stability at 37°C were shown in Table 1. There was no significant change in pH, particle size and EE for TSL, TSL diluted 50-fold with 5% glucose and TSL diluted 50-fold with MEM cell culture containing 10% FBS when kept at 37°C for 4 days; however, TSL diluted with cell culture media had a slight tendency of increase in diameter and decrease in EE. Almost no leakage of drugs from TSL happened during 4 days at 37°C. The short-term stability of the samples was also evaluated using Turbiscan Lab^®^ Expert. The variations of each sample in transmission or backscattering profiles over 4 days were shown in Fig 5. The results showed that variations of both transmission and backscattering were no more than 0.3% for all samples, and these changes were negligible, indicating that no apparent aggregation or sedimentation occurred in any of the samples during the culture period. It could be presumed from the results that TSL and its dilutions were stable and could be used after storage at body temperature for 96 h.

**Table 1:** Stability data of TSL and diluted TSL kept at 37°C.

Samples Detected	Time (day)	pH	Particle size (nm)	EE (%)
**TSL undiluted**				
	0	5.12	130.5±1.2	91.26±2.35
	1	5.08	132.2±0.5	90.71±1.97
	2	5.01	131.6±0.8	90.53±0.76
	3	5.10	133.0±0.3	90.32±2.17
	4	5.09	132.6±0.2	91.09±3.24
**TSL diluted 50-fold with 5% glucose solution**				
	0	5.13	132.9±0.7	91.74±2.81
	1	5.06	133.4±1.1	90.80±1.37
	2	5.10	132.3±0.9	91.46±1.65
	3	5.08	133.5±1.6	90.53±2.42
	4	5.11	130.1±0.4	90.48±2.39
**TSL diluted 50-fold with cell culture media containing FBS**				
	0	7.62	133.5±1.8	91.57±1.53
	1	7.64	133.1±1.1	90.48±1.47
	2	7.59	133.8±1.6	90.02±2.16
	3	7.68	134.2±0.4	89.52±0.67
	4	7.62	134.7±1.3	89.15±1.72

**Fig 6:**
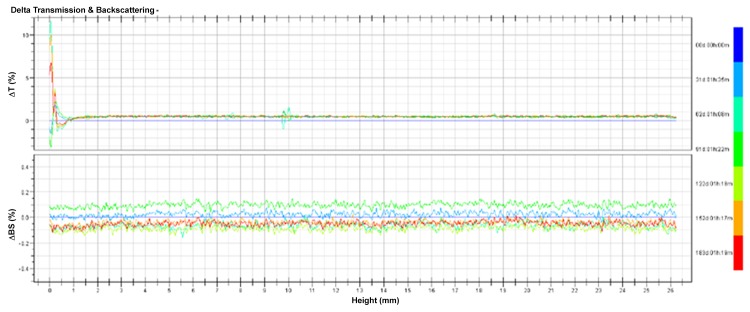
Variation profiles of transmission and backscattering for TSL in the long-term stability study.

The pH, particle size and EE of TSL kept at 4°C were also monitored for 6 months as shown in Table 2. There were no distinct changes for any of the indexes, including EE, suggesting that no drug leaked from TSL. The variations of no more than 1.0% in both transmission and backscattering for TSL over 6 months were exhibited in Fig 6. No apparent aggregation or sedimentation occurred in TSL during culture as the changes of both transmission and backscattering could be negligible. Taken together, these data suggested that TSL were stable at 4°C for 6 months.

**Table 2:** Stability data of TSL kept at 4°C.

Time(month)	pH	Particle size (nm)	EE (%)
**0**	5.11	130.6±0.4	91.23±1.57
**1**	5.10	132.4±0.6	90.26±1.72
**2**	5.05	132.3±0.5	90.31±0.83
**3**	5.12	131.9±0.9	90.67±1.87
**4**	5.14	132.5±1.2	90.84±2.16
**5**	5.16	133.4±0.8	90.53±2.35
**6**	5.12	132.1±0.7	90.25±1.87

Poloxamer has been introduced into several liposomal systems because its moderately concentrated solution is free-flowing at or below normal ambient temperature and is able to transform into gel at body temperature [33]. For example, it was reported by Zhang et al [27, 28, 30] that inserting poloxamer 188 into liposomes could strengthen the liposomal membrane and reduce both drug irritancy and *in vivo* precipitation. Poloxamer 188 was also mixed with DPPC as a liposomal membrane component to modify the surface of liposomes and prevent the phagocytosis of TSL by macrophages; however, the incorporation of poloxamer 188 into DPPC liposomes might destabilize the liposomal membrane [26]. It has also been reported that liposomes interiorly thickened with a great quantity of thermosensitive poloxamer had improved physical stability in comparison with conventional liposomes, and this enhancement of stability was considered to be related to the interior gelation of poloxamer [21]. Regretfully, drug release was seriously retarded owing to the addition of heavy poloxamer. In this paper, small amounts of poloxamer 188 were also added into the interior water phase to improve the stability of TSL. In these modified TSL, drug release was barely affected, and optimal TSL stability was acquired.

### *In vivo* anti-tumor efficiency

**Fig 7:**
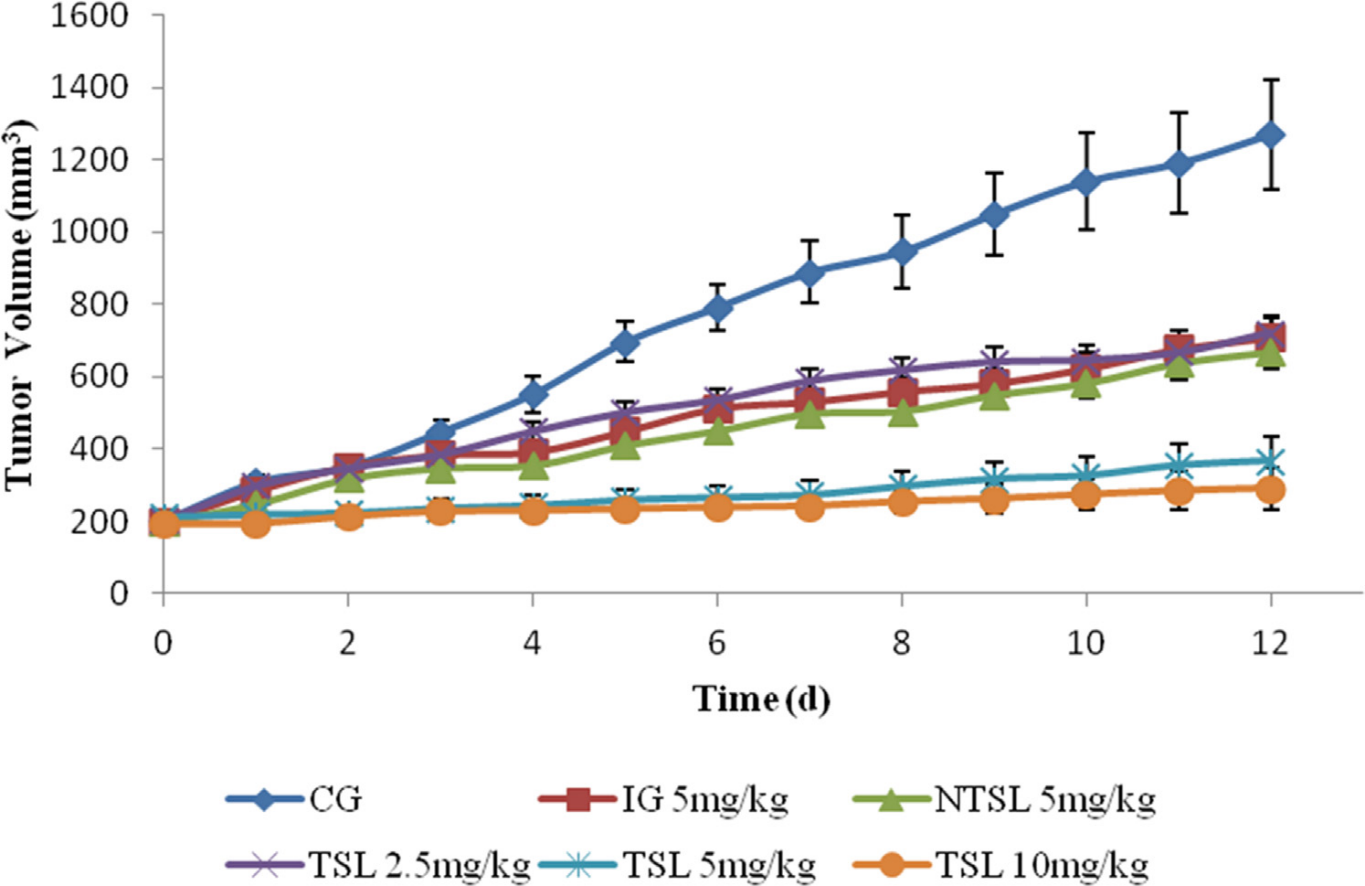
Tumor growth curves in male nude mice after treatment with 5% glucose solution (CG), 5 mg/kg OXP solution (IG), 5 mg/kg NTSL, 2.5 mg/kg TSL, 5 mg/kg TSL and 10 mg/kg TSL, respectively.

The anti-tumor activities of free OXP, TSL and NTSL were evaluated, and the results were shown in Fig 7 and Table 3. It was evident that the tumor volume for mice receiving 5% glucose solution as control increased rapidly during the whole 12 days and that the tumor growth for mice treated with OXP injection, NTSL and TSL was significantly inhibited. The anti-tumor activity of TSL at the dose of 2.5 mg/kg was almost equal to those of free OXP injection and NTSL at the dose of 5 mg/kg, which mean that the anti-tumor activity of OXP was significantly improved through the delivery system of TSL combined with HT. The anti-tumor activity of OXP in TSL was evidently dose dependent because tumor inhibition increased with improvement of the administration dose. The highest inhibitory effect of 81.3% achieved in the lab was observed in the TSL group that received a dose of 10 mg/kg.

**Table 3:** Volume, mass and inhibition rate of tumor (n = 12).

Group	Volume (mm^3^)	Mass (g)	Inhibition Rate (%)
**5% Glucose solution (CG)**	1270.6±150.6	0.91±0.49	-
**OXP solution 5 mg/kg (IG)**	710.2±58.5^a^	0.54±0.15^a^	40.6
**NTSL 5 mg/kg**	667.6±45.2^a^	0.51±0.11^a^	44.0
**TSL 2.5 mg/kg**	722.7±43.1^a^	0.50±0.31^a^	45.0
**TSL 5 mg/kg**	367.6±65.3^a^^b^	0.21±0.15^a^^b^	76.9
**TSL 10 mg/kg**	290.9±58.5^a^^b^	0.17±0.19^a^^b^	81.3

^a^*p*<0.05 compared with CG

^b^*p*<0.05 compared with IG.

HT has been applied successfully in the clinic to treat solid tumors, and HT combined with chemotherapy is recommended because cancer cells can’t be killed completely because of the gradual decrease in temperature from the center of the tumor to the periphery. A temperature-triggered drug delivery system is the most common combination of HT and chemotherapy. It is reasonable to expect increased accumulation of delivery carriers after localized HT due to elevated blood perfusion and vesicle extravasation, and TSL are considered to achieve the highest accumulation in all delivery carriers because of their rapid intravascular drug release in heated tumors [9, 31, 37]. In addition, the strong tumor inhibition of TSL in this paper was also attributed to high stability and limited drug leakage before TSL arrived at the heated tumor tissues.

**Fig 8:**
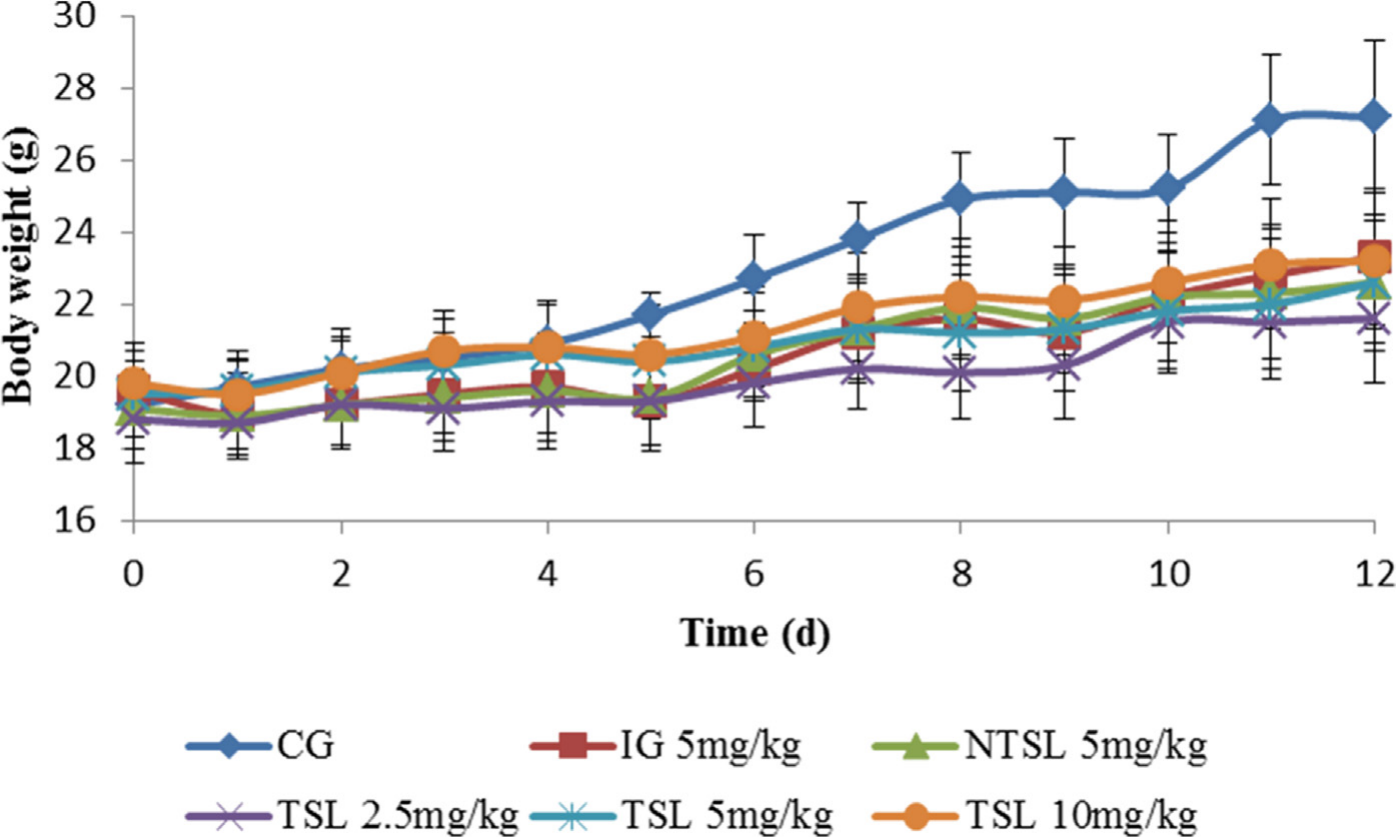
Body weight curves in male nude mice after treatment with 5% glucose solution (CG), 5 mg/kg OXP solution (IG), 5 mg/kg NTSL, 2.5 mg/kg TSL, 5 mg/kg TSL and 10 mg/kg TSL, respectively.

The body weight and health status of mice were also monitored during the whole treatment period to evaluate the toxicity of different formulations. As shown in Fig 8, there was a transitory decrease in body weight for mice administered drugs in any formulations, whereas this phenomenon was not found in mice administered 5% glucose solution, demonstrating that the weight loss might be caused by drugs. The mice treated with OXP solution manifested the largest reduction of body weight and appeared in low spirits, which mean that the most serious side effects happened in mice treated with OXP solution. A slight loss of body weight was also shown in mice administered NTSL or TSL with different dosages while there were almost no obvious changes in spirits for these mice. Therefore, it could be supposed that TSL had little toxicity and was safer than OXP solution and NTSL because it displayed very few side effects on mice and could be tolerated at higher dose.

**Fig 9:**
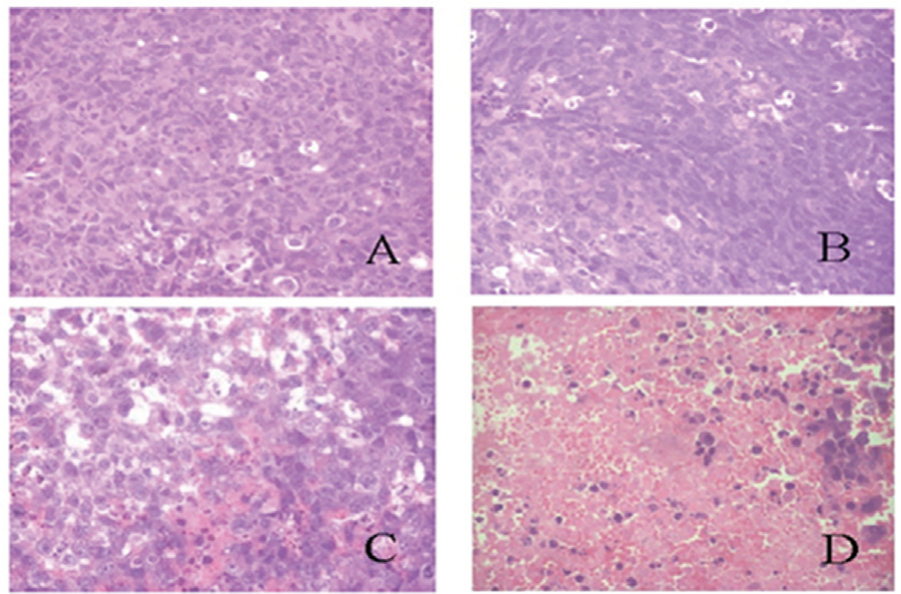
Photographs of murine LLCC tumor tissues stained with H&E. A: Treated with 5% glucose solution; B: Treated with OXP solution at the dose of 5 mg/kg; C: Treated with NTSL at the dose of 5 mg/kg; D: Treated with TSL at the dose of 5 mg/kg.

The histological transmutation of an excised tumor stained with H&E was shown in Fig 9. In the control group, tumor cells appeared integral, and their volumes were expanded and swollen. Nuclei with different sizes and coarse chromatin were also observed in the control group. Apparent transmutation of nuclei and pycnosis of some cells were displayed in the OXP injection group. Obvious pycnosis and slight necrosis of tumor cells were apparent in the NTSL group. Necrosis of most tumor cells, nuclear disappearance and dissolution after fragmentation, and homogeneous acidophilia were all observed in the TSL group at the dose of 5 mg/kg. So, it was evident that the degree of tumor transmutation was consistent with anti-tumor activity in the TSL group.

In conclusion, TSL showed more powerful anticancer ability than the injection solution and NTSL.

### OXP distribution in plasma and tumors

**Fig 10:**
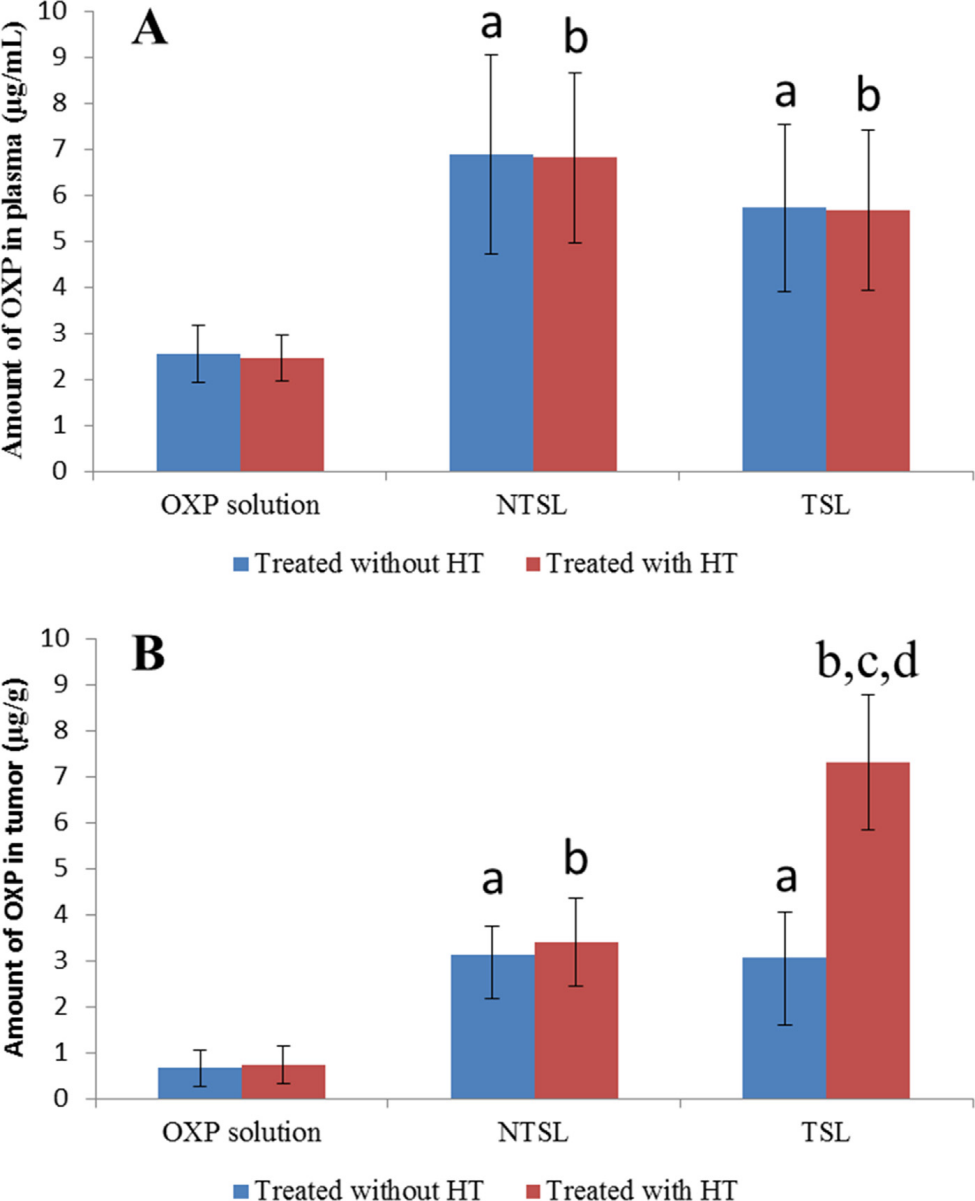
**OXP levels measured in plasma (A) and tumors (B) of mice treated with or without HT. The data are presented as the mean ± SD (n = 3).** a, *p*<0.05 versus OXP solution treated without HT; b, *p*<0.05 versus OXP solution treated with HT; c, *p*<0.05 versus NTSL treated with HT; d, *p*<0.05 versus TSL treated without HT.

To explain the targeting action of TSL combined with HT, the OXP concentrations in plasma and tumors of mice treated with OXP solution, NTSL or TSL combined with or without HT were determined (Fig 10). It was obvious that the plasma concentration of OXP in mice taken with NTSL and TSL was improved significantly compared with that in mice administered OXP solution regardless of HT, and there was no significant difference between plasma concentrations of OXP in mice given NTSL and TSL, regardless of HT. It seemed that the OXP concentration in plasma was hardly affected by HT for any of the formulations. The amount of OXP in tumors was increased significantly when OXP was encapsulated into either NTSL or TSL. HT greatly enhanced the amount of OXP in tumors in the TSL group, whereas HT had almost no effect on the distribution of OXP in tumors for both the OXP solution group and the NTSL group.

Relatively low efficacy had been reported for OXP injection, and it was believed that this low efficacy was due to its pharmacokinetic properties, such as high irreversible binding to plasma proteins, tissue proteins and erythrocytes [38]. For this reason, the encapsulation of OXP was expected to be a promising strategy to overcome these limitations [38]. PEGylated liposomes, which are also called sterically stabilized liposomes, can decrease drug clearance *in vivo* and increase drug accumulation in affected organ sites [38], and they remain the gold standard for long-circulating nanoparticles [39]. The higher plasma concentration of OXP for NTSL and TSL in this paper also verified lower binding of OXP with proteins or erythrocytes and longer circulating time *in vivo*. The higher tumor accumulation of OXP for both NTSL and TSL was perhaps related to the enhanced permeability and retention (EPR) effect [40]. Unfortunately, PEGylation significantly reduces the cellular uptake, endosomal/lysosomal escape and thereby, the efficacious drug release of the liposomes, which will interfere with the retention in tumor and antitumor efficacy of liposome-based drug delivery systems [9, 40]. So, it was necessary for PEGylated liposomes to achieve a higher drug accumulation at the target site. TSL combined with HT were used in this paper and more OXP accumulation in tumors was achieved from TSL-mediated drug delivery than from OXP solution or NTSL-mediated drug delivery. It could be illustrated to some extent that TSL made with poloxamer 188 were stable *in vivo* and that encapsulated drugs could be released immediately once TSL was combined with HT.

## Conclusions

OXP TSL with an entrapment efficiency of approximately 90% and complete drug release within 10 min after triggering were obtained through conventional TSL formulation combined with poloxamer 188. The high stability of these TSL *in vitro* was displayed for 4 days at 37°C and for 6 months at 4°C. The distinguished antitumor activity of TSL combined with HT was also certified. Taken together, the OXP TSL had an optimal release timing and efficiency, marked antitumor activity, and high stability. This system might provide an opportunity to improve the therapeutic index of OXP and offer an evocation for testing TSL in clinical trials.
